# On incoherent diffractive imaging

**DOI:** 10.1107/S2053273321007300

**Published:** 2021-08-27

**Authors:** Leon M. Lohse, Malte Vassholz, Tim Salditt

**Affiliations:** a Institut für Röntgenphysik, Universität Göttingen, Germany

**Keywords:** femtosecond studies, free-electron laser, correlated fluctuations, diffract-then-destroy, single particles, XFEL

## Abstract

Incoherent diffractive imaging (IDI) promises structural analysis with atomic resolution based on intensity interferometry of pulsed X-ray fluorescence emission. However, its experimental realization is still pending and a comprehensive theory of contrast formation has not been established to date. Explicit expressions are derived for the equal-pulse two-point intensity correlations, as the principal measured quantity of IDI, with full control of the prefactors, based on a simple model of stochastic fluorescence emission. The model considers the photon detection statistics, the finite temporal coherence of the individual emissions, as well as the geometry of the scattering volume. The implications are interpreted in view of the most relevant quantities, including the fluorescence lifetime, the excitation pulse, as well as the extent of the scattering volume and pixel size. Importantly, the spatiotemporal overlap between any two emissions in the sample can be identified as a crucial factor limiting the contrast and its dependency on the sample size can be derived. The paper gives rigorous estimates for the optimum sample size, the maximum photon yield and the expected signal-to-noise ratio under optimal conditions. Based on these estimates, the feasibility of IDI experiments for plausible experimental parameters is discussed. It is shown in particular that the mean number of photons per detector pixel which can be achieved with X-ray fluorescence is severely limited and as a consequence imposes restrictive constraints on possible applications.

## Abstract 2

Starting from a simple model of stochastic fluorescence emission, a theory is derived of contrast formation and signal-to-noise ratio for incoherent diffractive imaging; its feasibility for plausible experimental parameters is discussed.

## 1. Introduction   

X-ray diffraction capitalizes on the fact that microscopic signals of scattered waves add up coherently and form a macroscopic interference pattern which can be captured by X-ray detectors in the far-field. Hence, within a volume defined by a coherence length of the radiation, the signal is enhanced by constructive interference proportional to the square of the scattering centers 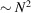, most notably in the forward direction and, for diffraction from crystals, at the Bragg peak positions. Moreover, even for distances beyond the coherence length, the diffraction signal scales linearly with the interaction volume. Modulating the scattering intensity as a function of the outgoing wavevector 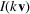 with wavenumber *k* and unit direction vector 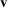, the measurable far-field interference pattern thus encodes the spatial Fourier transform of the ‘scattering length density’ 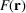 on atomic scales. Since the scattering process is coherent, the difference between the outgoing 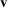 and incoming wavevector 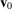 determines the phase shifts between scattering centers, and in the kinematical scattering regime the intensity is proportional to the structure factor 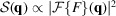 as a function of the scattering vector 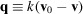.

Notwithstanding the abiding importance of coherent diffraction, the small cross section of Thomson scattering limits structural analysis at the level of single small crystallites or even single molecules. This has raised a desire to make better use of the much higher cross section for photo-electric absorption, not only for spectroscopy, but also for a generalized diffraction method. Depending on photon energy 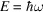 and atomic number *Z*, the ratio of cross sections for photo-electric absorption and elastic (Thomson) scattering can easily result in hundreds of photons being absorbed per coherently scattered photon. Photo-ionization then leaves an excited atom with an inner-shell vacancy behind, from which a sizeable fraction decays via emission of an X-ray fluorescence photon. Fluorescence emission was long perceived not to convey any information about the microscopic sample structure, due to the random nature of the emission with independent phases.^1^ Correspondingly, one would expect a random speckle pattern in the far-field, encoding not only the path length differences due to structure but also the unknown random phase differences. Yet, all structure in the far-field pattern ‘averages out’ because the *coherence time* of X-ray fluorescence is generally on the order of 1 fs, and all measurements are significantly longer than the coherence time.

However, as observed by Classen *et al.* (2017 ▸), the pulse length of modern XFELs (X-ray free-electron lasers) on the order of a few up to a hundred femtoseconds provides an intrinsic time gating for the fluorescence emission. Although not yet quite as short as the coherence time of X-ray fluorescence, the pulse lengths might just be short enough to leave some structural information in the fluorescence. In the work of Classen *et al.* (2017 ▸), a simple time-independent quantum-mechanical model was used to show that the two-point correlations of the fluorescence intensity are in fact proportional to the very same structure factor 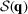 which emerges in coherent scattering plus a constant offset. They hence proposed a method to extract spatial information from incoherent diffraction patterns, which they termed incoherent diffractive imaging (IDI) in analogy to coherent diffractive imaging (CDI) (Miao *et al.*, 1999 ▸, 2015 ▸; Chapman *et al.*, 2006 ▸). The underlying principle, intensity interferometry, goes back to the work of Hanbury Brown & Twiss (1956 ▸), where it was used to measure the angular diameter and separation of stars. However, we are not aware of any successful realization of IDI with atomic resolution, to date.

A closely related experiment, exploiting two-point intensity correlations of X-ray fluorescence, was reported by Inoue *et al.* (2019 ▸). The authors used the correlations in the fluorescence from a thin copper foil, excited by XFEL pulses from SACLA (SPring-8 Angstrom Compact free electron Laser), to infer the duration of the exciting pulse and spatial extent of the focal spot. Unlike IDI, however, which aims for 3D *imaging* with atomic resolution, the latter experiment extracted only the spatial extent of the scattering volume with a resolution just below 1 µm, and thus served mainly for characterization of the pulse itself. Similarly, two-point intensity correlations have been studied analytically as a means to deduce the source size as well as the detector point-spread-function (Gureyev *et al.*, 2017 ▸). While the experiment of Inoue *et al.* (2019 ▸) is hence still far from a realization of IDI with atomic resolution, it is conceptually similar, since in both cases structural information is extracted from two-point intensity correlations of hard X-ray fluorescence. Further, two-point and higher-intensity correlations of incoherent diffraction data have also been exploited to reconstruct 2D test structures imaged by FEL (free-electron laser) pulses (Schneider *et al.*, 2017 ▸). They are thus prototypical for an emerging class of experiments, where the information is not contained in the mean of the experimental data (which is homogeneous), but in its dependency structure.

The concept of IDI and the absence of its experimental demonstration have also sparked theoretical investigations. Ho *et al.* (2020 ▸) discussed the coherence time of hard X-ray fluorescence following the excitation by intense XFEL pulses. More recently, Trost *et al.* (2020 ▸) discussed the signal-to-noise ratio (SNR) of IDI, using a simple time-independent wave-optical model. Their model considers a set of ‘emitters that emit monochromatic spherical waves with random relative phases’ and includes the photon (detection) statistics of a pixel detector. Based on their model, they derive and simulate the scaling of the SNR of the two-point photon correlations as a function of several external parameters, such as the mean number of counts per pixel, the number of emitters and the number of modes. They also give an analytical expression for the number of modes as a function of the coherence time, the duration of the excitation pulse and the polarization state. Moreover, they mention that as the pixel size is increased, the number of modes increases. However, they do not explicitly treat this ‘speckle sampling’ effect, arguing that it can be considered by an appropriate adjustment of the parameters. Similarly, they discuss only qualitatively how ‘large crystals’ can lead to an increased number of modes, when the linear extent of the scattering volume is greater than the coherence length of the emissions, but do not quantify this effect. They conclude, in agreement with the seminal work of Classen *et al.* (2017 ▸), that, under optimized conditions, ‘IDI may offer utility in structure determination of single molecules at X-ray FELs’ and that ‘IDI could potentially provide element-specific structural information to complement weak coherent scattering’ (Trost *et al.*, 2020 ▸).

Here, we ask to what extent we can translate our intuition from the kinematic and dynamic theory of coherent diffraction to incoherent diffraction? In particular, can we develop a quantitative understanding of contrast formation in incoherent diffraction, including the effects that were mentioned qualitatively in the works of Classen *et al.* (2017 ▸) and Trost *et al.* (2020 ▸) in a self-contained way?

To answer these questions, we develop a *time-dependent* probabilistic model for incoherent emissions following a short excitation pulse that accounts for the geometry of scatterers and detector. We treat the fluorescence emissions with fully specified (self-) coherence functions and explicit emission and propagation times. Since two emission events may have lost temporal overlap when reaching the detector, in particular for extended samples, the effective contrast can be severely degraded. We derive detailed estimates for the contrast in terms of a few parameters, including coherence time, sample extent, as well as the number and relative strength of emission lines. In particular, we can show that the contrast is inherently limited and derive a universal upper bound on the photon yield and correspondingly on the SNR.

The text is structured as follows. In Section 2, we describe the model, compute some statistical properties in terms of the radiated energy, and discuss contrast formation in the two-point correlations. We subsequently extend this model in Section 3 to account for quantized photodetection and derive an expression for the SNR. In contrast to the statistical treatment in the work of Trost *et al.* (2020 ▸), we treat the coincident measured counts as statistically dependent random variables and give rigorous lower bounds on the noise level and thereby rigorous upper bounds on the SNR. In the course of Section 4 we discuss different contributions to the contrast with special emphasis on the geometrical implications of the finite coherence time, propagation of the excitation pulse, and non-negligible size of the scattering volume. Section 5 is concerned with spatial sampling of the correlation signal and the implications of finite-sized detector pixels. In particular, we quantify, using rigorous upper bounds on the contrast, how the contrast decreases with increasing angular pixel size and size of the scattering volume, as was previously mentioned qualitatively in the works of Trost *et al.* (2020 ▸) and Classen *et al.* (2017 ▸). In Section 6 we estimate and show how the constraints imposed by the contrast relations inherently limit the photon yield. We use these estimates to discuss the feasibility of experiments in Section 7. Section 8 summarizes and concludes our findings.

## 2. A probabilistic model for incoherent emissions   

We develop a simple probabilistic model to describe the random incoherent emissions from an ensemble of emitters, following excitation by a short pulse. A time-dependent description is necessary to capture the loss of contrast due to the finite coherence time in a closed form. We use this model to derive statistical properties of the energy that is radiated during each pulse and show how the structure factor of the emitter distribution enters the two-point intensity correlations. The main symbols used throughout the article are listed in Appendix *C* [App appc] for reference.

### 2.1. Setting   

**Figure 1:**
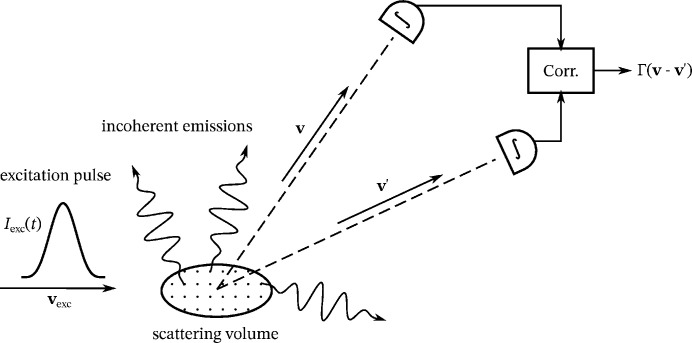
Sketch of the experimental principle based on coincident detection of incoherent emissions. The single-pulse two-point correlations 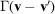 contain the structure factor of the emitter ensemble.

Let us consider the following experimental setting, as sketched in Fig. 1 ▸. An ensemble of atoms is irradiated by an ultrashort excitation pulse that excites a sizeable number of them. Some fraction of the excited atoms subsequently undergoes a radiative transition and each emits a fluorescence photon. These photons are registered by (energy-sensitive) pixels of a 2D pixel array detector. The average radiated energy is isotropic if we neglect secondary interactions with the sample. However, the equal-pulse two-point intensity correlations Γ contain a structural signal of the emitter ensemble.

IDI, as originally proposed, uses pulses from an XFEL that produce inner-shell vacancies due to photo-absorption. There, the majority of the emitted photons stem from radiative transitions to the *K* shell, most frequently the 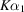 and 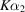 transitions. The model we describe, however, neither depends on the nature of the excitation pulse nor on the particular type of transitions, and should therefore be equally applicable to other spectral ranges, in particular the optical regime, and even other kinds of incoherent emissions. We formulate the theory as generally as possible, referring to the parameter range of *K*α radiation only for examples.

Significant correlations can only be observed when the pulse duration (implementing the time gating) is comparable to the coherence time of the emitted light. The coherence time of inner-shell fluorescence radiation is on the order of femtoseconds, which is significantly shorter than the time resolution of any conceivable detector. We thus assume that only the total energy, deposited over an entire pulse, is registered experimentally.

Although emissions of individual photons are typically discussed in a quantum-mechanical formulation, a large number of emissions can be conveniently described semi-classically. Interference in two-point intensity correlations from a pair of incoherently excited atoms is discussed for example in the book by Agarwal (2013 ▸), ch. 14. There it is shown that the emerging interference fringes have 100% visibility, which is a clear sign that the underlying process is non-classical (Mandel, 1999 ▸). However, the visibility gradually decreases with increasing number of emitters 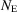 and asymptotically approaches 50%. In fact, Classen *et al.* (2017 ▸) have calculated that the interference patterns produced, respectively, by single-photon emitters (SPE) and thermal light sources (TLS) converge with one another with 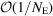. It thus seems plausible that the interference from a large number of emitters can be approximated well with a semi-classical description, but special care has to be taken when discussing two-photon contributions.

### 2.2. Basic assumptions   

**Figure 2:**
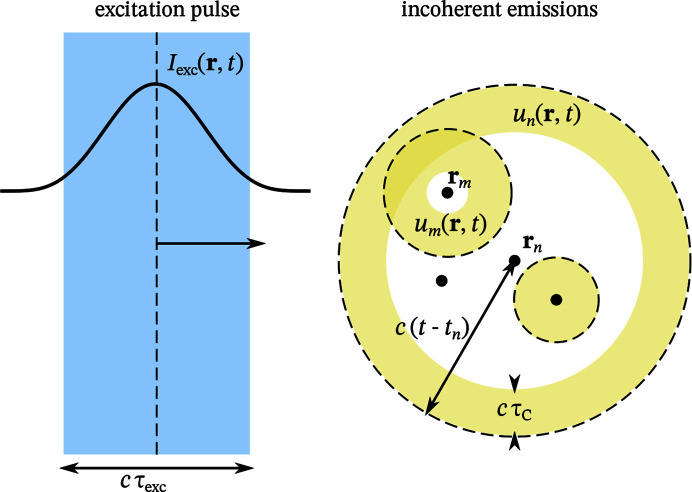
Sketch of the probabilistic model. The emitters at fixed positions 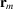 randomly emit spherical waves with finite coherence time 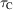. The moment of emission is random and proportional to the instantaneous intensity of the excitation pulse, which is assumed to be a traveling wave with pulse duration 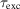.

First, we discuss the emissions as randomly parameterized classical electromagnetic waves (see Fig. 2 ▸). Later, in Section 3, we additionally include quantized photodetection, following the book of Goodman (1985 ▸). We initially suppose that the fields are perfectly polarized to simplify the notation. The unpolarized nature of fluorescence is taken into account in Section 4.3. Let 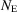 be the number of individual emitters and let 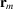 for 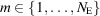 denote their positions. We assume the system to be stationary in the sense that each pulse has the same initial conditions and can be represented as a realization of the same statistical ensemble.

We make the following assumptions:

(i) The event in which a specific atom with index *m* emits a photon follows a Bernoulli distribution with probability 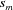. We associate the random variables 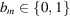 with the emission of a photon.

(ii) The photon energy 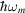 takes values from a finite set of discrete transition energies (emission lines) 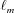 with probabilities corresponding to the relative line intensities.

(iii) The emission times 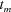 are randomly distributed with probability density proportional to the cycle-averaged local intensity 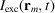 of the excitation pulse (the lifetime of the excited state is described by the time evolution of the field amplitudes) at position 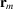.

(iv) The emissions are independent, such that the random variables characterizing the emissions, 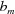, 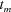 and 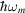, are mutually independent.

(v) The emissions can be described by outgoing spherical waves.

Let 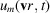 be the complex-valued analytic signal associated with the electromagnetic disturbance due to the emission from the *m*th atom. The normalized vector 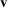 denotes the observation direction relative to the center of the scattering volume. Let 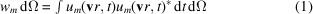
denote the energy flow of 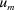 into the infinitesimal solid angle 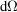 at some point 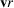 in the far-field. The total electromagnetic disturbance at position 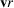 due to all the emissions can be written as 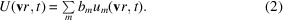
The total energy flow through 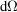 becomes 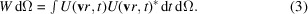



### 2.3. Correlations   

These basic assumptions suffice to significantly simplify the average intensity and two-point intensity correlations. The expectation value of *W* can be expressed as 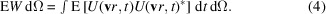
Inserting (2) and exploiting the independence of the individual emissions show 
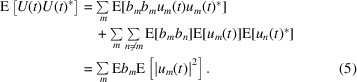
Here, we have used 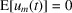 and 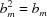. Performing the time integral and inserting 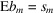 yields 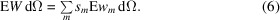



Next, we calculate the two-point correlations of the intensity. Consider the energy flow into the direction 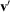 through the solid angle 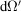 as a second observable. We are interested in the correlation of 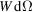 and 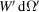, expressed as 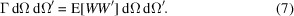
Importantly, the random variables that appear are identical for both observation directions. We obtain 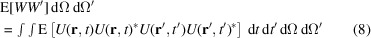
and 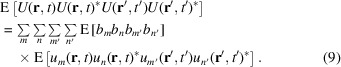
Here, we use the notation 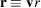 and 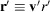. Since the emission times are mutually independent and 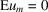 as well as 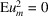, only certain combinations of the indices survive the expectation value: 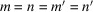, 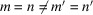 and 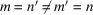. Consequently, (9) becomes 
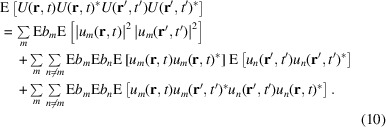
Performing the time integration yields 
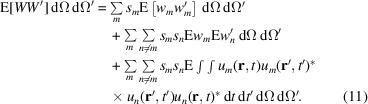
To evaluate the remaining integrals, we express the 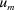 as 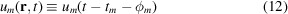
with geometric phases 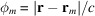. The integral in (11) can then be expressed as 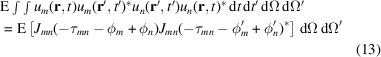
with 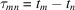 and interference terms 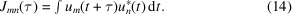
In the far-field we have 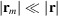 such that the usual far-field approximation gives 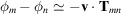 with 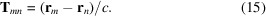
In the following we suppress the solid-angle differentials.

To further evaluate these interference terms (14), we require additional assumptions on the temporal structure of the individual emissions. We assume that the emissions produce electromagnetic signals that are characteristic for the involved atomic energy levels (emission line 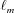) up to the spatial origin 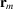 and emission time 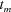. More precisely, we parameterize the waves in terms of their respective temporal and spatial origin and their emission line 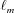 such that 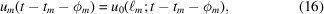
where 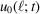, for each emission line 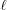, describes an outgoing spherical wave. Inserting this into (14), we obtain 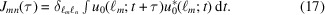
We have assumed that signals with 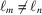 do not interfere because the emission lines do not overlap.

### 2.4. Spectrum and self-coherence   

It turns out that (17) is fully determined by the complex degree of coherence (CDC) of *U*, which in turn is fully determined by the (phase-less) spectrum of *U*.

For simplicity, we ignore the spatial dimensions and also consider the simplest situation of only a single contributing emission line 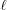. Then, we may express the self-coherence at time delay τ of the total signal *U* as the ensemble average (Mandel & Wolf, 2015 ▸):
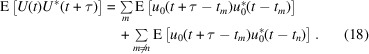
Here the expectation value is taken over the time offsets 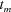 which are distributed over some time interval longer than the coherence time. Since 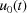 oscillates rapidly with some central frequency 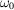 and the time offsets are mutually independent, each non-diagonal term must vanish. The diagonal terms become 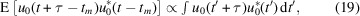
for 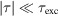. Here the duration of the excitation pulse 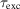 quantifies the width of the distribution of 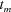. Then, also the self-coherence (18) is proportional to 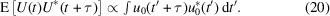
Normalizing the left-hand side gives by definition the CDC 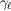 associated with the emission line 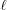, such that 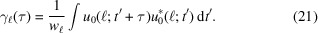
The temporal autocorrelation of 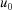 is hence directly related to the macroscopically accessible CDC of an isolated emission line. Importantly, equation (21) implies that the temporal autocorrelation of 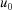 is fully determined by 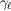, which is determined by the coherence time 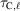 and the line shape.

### 2.5. Interference terms   

Substituting (21) in (17) yields 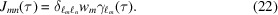
The CDC factorizes into a rapidly oscillating part and an envelope, such that 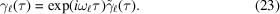
The envelope is real for symmetric line shapes but may take negative values in general. However, for Gaussian and Lorentzian line shapes, it is restricted to non-negative real values. It can hence be expressed as 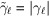. In particular for Lorentzian line shapes, we have 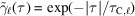 where 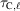 is the coherence time. Inserting the factorized 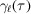 yields 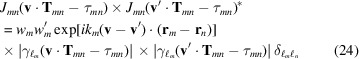
where 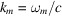.

Suppose that the emissions are spectrally filtered so that only photons from a relatively narrow energy band are registered in the detectors. More precisely, suppose that 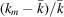 is very small for all *m*, where 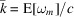 is the mean wavenumber of the involved frequencies. In the same sense suppose that the pulse energies are approximately equal, *i.e.* 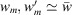. The expectation value of (24) can then be written as 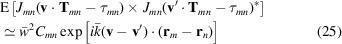
with 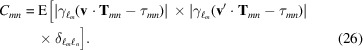
The coupling coefficients 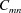 take values in the interval 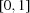. Note that their diagonal elements are 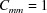 and that 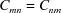 as can be readily seen. From here on set 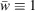 and 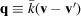 to simplify the notation.

After inserting (25) into (13), (11) finally yields 
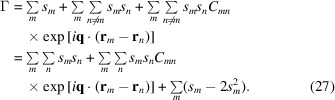
Comparing this expression with the result of Classen *et al.* (2017 ▸), which was derived with a time-independent quantum-mechanical description of SPE, shows two differences. First, their model is time independent and as such implicitly assumes 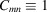. Second, our classical formulation fails to reproduce that the diagonal terms, corresponding to coincident detection of the same atomic emission, must vanish. However, since the diagonal terms scale with 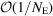 relative to the other terms, they can be neglected for large 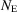 and the two expressions converge.

Using 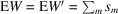 and neglecting the single sum simplifies the correlation to 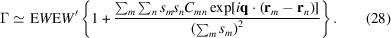
The right-hand side of (28) consists of two parts: a constant background 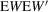 and a structural signal 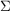 = 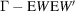. It follows that 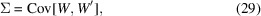
using 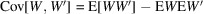.

### 2.6. Effective contrast and structure factor   

The two-emitter contributions in (28) are individually attenuated by the coupling coefficients 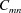. Although these coefficients could be computed individually for a specific model, it is instructive to consider their global average, 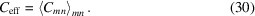
Using the approximation 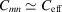 decouples the contributions of the individual emitters in (28) and the right-hand side simplifies to 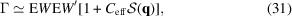
with 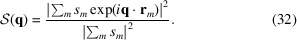
Equation (32) resembles the definition of the *structure factor* defined in the context of elastic scattering, although the coefficients have a different meaning.

The sums can be expressed as integrals. Defining the (unit-less) emitter distribution 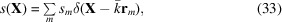
we can write the structure factor (32) as 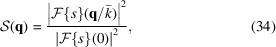
where 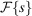 denotes the 3D Fourier transform of 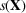. We scaled the emitter distribution with the wavenumber 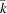, because it is a property of the emission line and hence determined by the emitters. We will mostly use the unit-less scattering vector, defined as 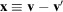, in place of 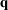. The two are related by 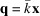 so that 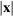 probes a length scale of 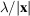. We will use the two notations 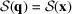 synonymously.

Equation (31) is equally applicable for 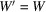, which provides the variance 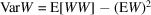. Using 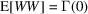 and 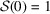, we read off that 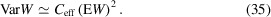
For clarity and later reference, we rewrite (31) more explicitly to include the two observation directions and their solid-angle differentials as 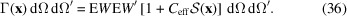



### 2.7. Comparison with elastic scattering   

Compare (36) with the elastically scattered intensity, which can be written as 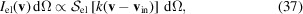
where here 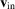 is the propagation direction of the *incoming* beam and *k* the wavenumber of the incoming and scattered beam. Recall that the elastic structure factor 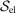 is defined as in (32); however, instead of the emission probabilities 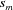, the coefficients are interpreted as atomic form factors.

There are two profound formal differences between (37) and (36) besides the dependence on one and two directions, respectively. First, (36) contains constant (unit) offset. Second, the signal is attenuated by the constant 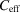 quantifying the contrast of the structure factor to this constant background. The correlation function thus contains a constant *intrinsic background* and the *intrinsic contrast* of the structural signal to this background is always less than unity. The coherently scattered intensity on the other hand has no intrinsic background and therefore no intrinsic contrast limitations. Only additional processes that contribute to the intensity form a background and limit the contrast. This extrinsic contrast, however, is not limited to unity.

## 3. Photon statistics and noise   

The goal of this section is to estimate the noise level of measurements of Σ. Apart from the fluctuations of the classical energies *W*, which are caused by the randomness of the emissions (source noise) [Trost *et al.* (2020 ▸) call this contribution phase noise, because it originates from the random phases in their model], additional fluctuations emerge due to the quantization of the electromagnetic field or the detection process (shot noise). In fact, in the low-photon regime, this second source of noise is the bigger contribution to the noise level. Importantly, it is independent of the signal level of the correlation.

### 3.1. Statistics at a single observation point   

We follow the semi-classical formalism described in the textbook of Goodman (1985 ▸), starting by repeating some fundamentals for reference purposes. Let *K* be the number of counts registered by the detector at observation direction 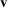 and covering the solid-angle differential 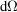. The conditional distribution 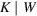, conditioned with the energy *W* entering the detector, is Poissonian with parameter 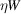, where η is the quantum efficiency of the detector. (Here we include the photon energy 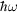 in the definition of the quantum efficiency.) The expectation value and variance of the (unconditional) *K* can be expressed in terms of moments of *W* as 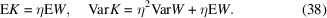
Inserting (35), which gives 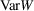 in terms of 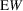 for the model discussed in Section 2, we obtain 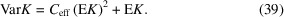
Equation (39) gives the fluctuations of the number of counts measured with a single detector as a function of the mean counts. The fluctuations have two contributions: the source noise, 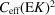, and the shot noise, 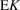. As we are going to see in Section 6, the mean count numbers that can be expected are usually well below 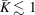. In this regime, the shot noise strongly dominates the source noise.

### 3.2. Count-correlations   

The situation is more complicated for coincident measurements at two or more points, because the emissions into the two respective directions are correlated. We can, however, assume that the two detectors do not influence each other, such that the conditional random variables 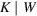 and 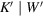 are in fact independent. Under this assumption we have that 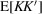 = 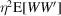 and similarly 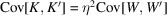 (Goodman, 1985 ▸). As a result, the correlations and covariances of the two classical energies and the two photon counts are related simply by a constant factor. In particular, we can define the signal as 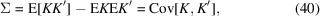
and use the expressions for 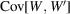. Note that (40) assumes 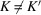, because otherwise the two conditional distributions are no longer independent. For 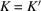 we get 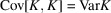 which includes a shot-noise term that is absent in (40) (Singer & Vartanyants, 2013 ▸).

### 3.3. Fluctuations in the count-correlations   

The covariance on the right-hand side of (40) can be expressed as 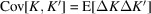, where 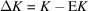 and 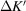 alike. We would like to calculate the second central moment, 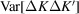. Unfortunately, there is no simple identity relating this expression to moments of *W* and 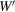. A simple but rather lengthy calculation (see Appendix *B* [App appb]) shows that 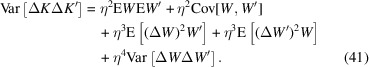
In particular, since all terms on the right-hand side are non-negative, we conclude that 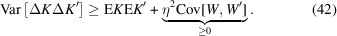
This proves that the variance is greater than or equal to the product of the shot noise of two individual (independent) intensity measurements, irrespective of the joint statistics of *W* and 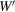. It is obvious that the other terms in (41) will contribute significantly for higher average counts 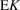, so that the variance scales differently. However, this high-photon regime is of little relevance here due to the low photon numbers that can be expected (see Section 6). For further discussion of this high-photon-count regime, see the work of Trost *et al.* (2020 ▸).

### 3.4. Measurements and SNR   

We now turn our attention to the SNR for estimates of the covariance Σ. We first specify, for reference, how to compute Σ from measured coincident realizations of *K* and 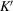. Suppose that 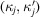 for 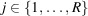 are *R* independent (coincident) realizations of 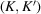. Define the sample mean as 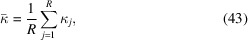
and 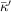 alike, and set 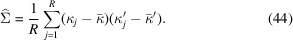
Then 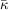 converges to 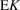 and 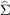 converges to 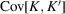 for increasing *R*. The SNR of 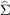 can be defined as 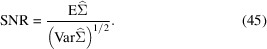
Using 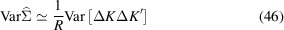
and inserting 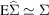 we find that 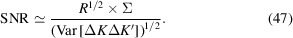
Inserting (42) and 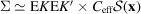, and assuming isotropic radiation with 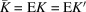, we conclude that the SNR is bounded by 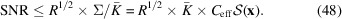



Recall that (42) bounds the variance by the shot noise of the intrinsic background, which results in a strict upper bound on the SNR. For higher photon counts, however, the source noise may exceed the shot noise so that (48) overestimates the SNR. In fact, for increasing 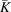, the SNR *saturates* at 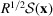 (Trost *et al.*, 2020 ▸).

When only a single pair of detectors is used then the number of independent realizations *R* is given by the number of pulses 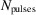. In practice, however, it makes sense to use a pixel array detector and acquire a large number 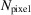 of measurements simultaneously. Since not all possible pairs of pixels correspond to distinct scattering vectors, every frame provides multiple realizations for the same 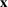 simultaneously. The multiplicity 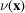 corresponding to the scattering vector 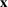 decays from 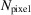 for 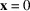 to 1 for large 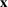, where its exact shape depends on the chosen discretization and the detection geometry. Note that these simultaneous realizations are in general not independent. Even if the individual intensity measurements were independent (if the coincident intensity measurements were strictly independent, their correlation, and thereby the signal, would vanish), then the individual terms in (44) would still be correlated, because some 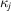 appear more than once (Trost *et al.*, 2020 ▸). Nevertheless, for the purpose of an optimistic upper bound on the SNR, we can use 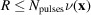 and implicitly count the simultaneous realizations as if they were independent.

Solving (48) for 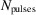 gives the minimum number of pulses required for a certain target SNR and a given signal level: 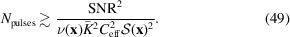
The main results of this section are (48) and (49), giving an optimistic bound on the relation between the SNR and the number of pulses. Although we derived the SNR only for a specific method of estimating the two-point covariances, which we do not claim to be optimal, the result illustrates a general characteristic: for low photon counts, the noise in the estimated covariances is dominated by the shot noise of the individual measurements, which is independent of the signal level. As a result, the signal level does not cancel out and the SNR is linearly proportional to the signal level. These exponents, together with the scaling of contrast and photon counts, sensitively govern the achievable SNR for different experimental settings, as we will discuss in Section 6.

## 4. Contrast estimates   

Since the contrast 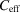 is a crucial parameter in (36) and (48), it seems appropriate to discuss it in more detail. We make some simplifying assumptions to factorize 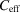 and discuss the main constituents individually. Each can be estimated from a few parameters, such as the duration of the excitation pulse, the spatial extent of the scattering volume, and the spectrum of the emitted radiation.

Suppose for simplicity that all involved emission lines have the same coherence time 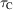 and therefore that 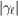 is identical for all emission lines. Then, the coefficients (26) factorize into a spatiotemporal (ST) and spectral (L) factor, 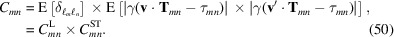
Here we dropped the index 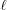 since all 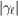 are alike.

Equation (50) gives an approximate expression for the coupling coefficient between two specific emitters. The macroscopic contrast 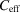, however, is determined by the *average* coupling constant between all pairs of emitters. Assuming that the spectral factor is constant for all pairs of emitters allows one to take the averages separately, so that 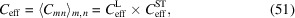
with 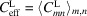 and 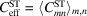.

### 4.1. Spectral overlap   

We discuss the spectral contribution first. Suppose, for simplicity, that all emitters have identical emission spectra, consisting of discrete emission lines with relative intensities 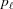. In that case, the first contribution to the coupling coefficients becomes 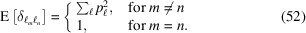
The factor therefore does not depend on *m* and *n*, but differs for diagonal (identical emitter) and non-diagonal (distinct emitters) contributions. Since there are only 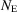 diagonal terms, but 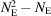 non-diagonal terms, only the latter contribute significantly to 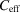. In particular, the *K*α line splits into the 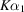 and 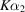 lines with relative intensities of 2/3 and 1/3, so that 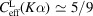.

### 4.2. Spatiotemporal overlap   

A pair of emissions contributes to the structural signal only when the two emissions arrive within the coherence time at each pixel. This temporal overlap is purely governed by the duration of the excitation pulse when the scattering volume is smaller than the coherence length. For larger scattering volumes, however, the finite-speed propagation of the excitation pulse and of the emissions also enter the picture, and we have to consider the combined temporal and spatial overlap.

The overlap 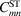 could be approximated analytically for certain symmetric configurations; however, covering all possibilities would be lengthy and cumbersome. Instead, our strategy is to write down the full expression and to derive some complementary upper bounds for it. In this way, we can include all configurations with little effort and give rigorous estimates on the maximum achievable contrast.

The factor 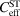 is governed by the emitter locations 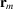 and the probability distribution of the emission times 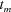. We write the probability density function of the temporal separation 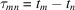 as 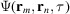. The probability density of 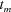 is proportional to the intensity of the excitation pulse 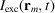, by assumption, so that 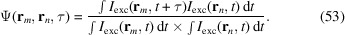
The spatial diagonal 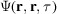 is the temporal autocorrelation of the excitation pulse, which, neglecting dispersion, does not depend on 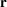. For brevity we write 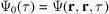.

We model the excitation pulse as a dispersion-less plane wave with propagation direction 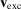, group velocity *c* and duration 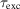. Since a plane wave can be written as a function of a single argument, 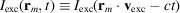, a simple shift in the integration variable of (53) shows that the cross-correlation can be expressed as 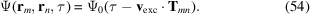
Note that 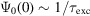 and 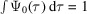.

The expectation value can be expressed as 
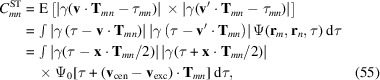
where 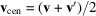. For the last identity, we have substituted (54) and shifted the integration variable. First, we focus on the purely temporal components of the overlap, corresponding to a small scattering volume. Suppose that all emitters lie close together such that 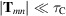 and 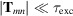. Under that assumption (55) simplifies to 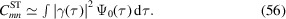
Importantly, this quantity is position-independent. We can give upper bounds to (56) using the inequality 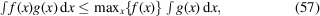
which holds for non-negative functions *f* and *g*. Noting that 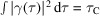, we obtain 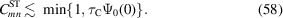
This shows that the contrast is independent of the emitter geometry and hence ‘pulse limited’, for small scattering volumes.

**Figure 3:**
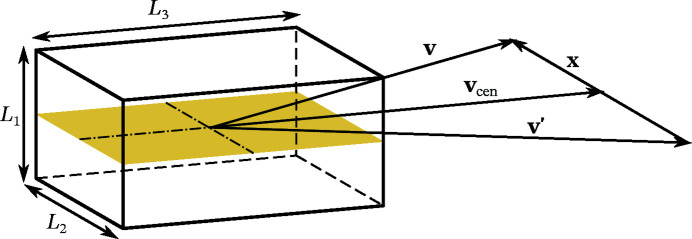
Consider a collection of emitters that is homogeneously distributed in a cuboid scattering volume with edge lengths 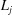. The edges of the cuboid are aligned with respect to the scattering vector 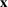 and 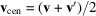.

Larger scattering volumes, on the other hand, do affect the contrast. Consider a cuboid scattering volume, aligned with the observation directions as shown in Fig. 3 ▸. Assuming a homogeneous emitter distribution, we can calculate the average over all emitter pairs. The average can be expressed as 
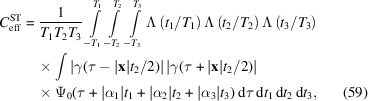
where 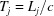 are the propagation durations, 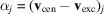 are the respective projections to the coordinate axes, and 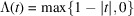 is the unit triangle function. Note that the dependence on 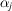 can be neglected when 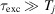 and 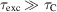 because then 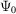 is essentially independent of its argument. Exchanging the order of integration, we obtain 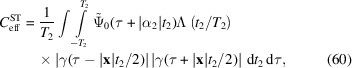
with 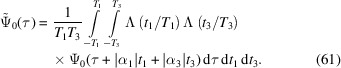
Note that (60) reduces to (58) in the limit of small 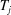. First, consider the effect of 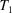 and 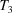 on 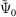. Using (57) on both integrals and extending the bounds to infinity, we obtain 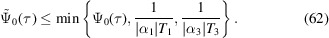
Next, applying (57) on the integral over 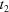 in (60) shows that 
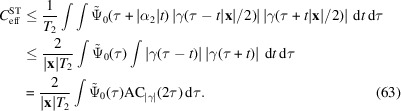
For the second inequality we have set 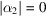, which does not decrease the value of 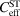. Here 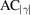 denotes the temporal autocorrelation function of 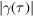, 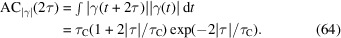
For the second identity we have used 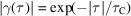, corresponding to a Lorentzian line shape. The time integral reads 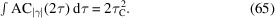
Inserting (62) into (63), using (57) to bound the integral over 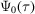, and then inserting (65) yields 
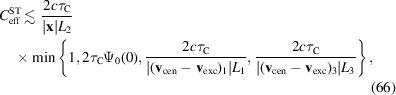
which is the equivalent of (58) for larger volumes and large scattering vectors 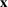.

Equation (66) is only useful for 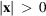. Going back to (63), we find another inequality for 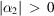. More precisely, (63) implies 
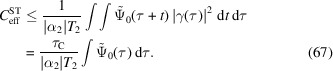
Here we have set 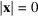, which only increases the value of the integral. Inserting 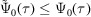 yields 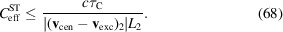
This shows that even for small scattering vectors, the contrast can be significantly below the pulse-limited contrast, when the detector is not placed in the forward direction with respect to the excitation pulse. This effect is most pronounced with 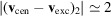 in a ‘back-scattering’ geometry but also enters with 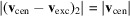 in the 90° geometry that has been proposed by Classen *et al.* (2017 ▸) and is depicted in the work of Trost *et al.* (2020 ▸, Fig. 1).

In summary, we have discussed that for small scattering volumes (*i.e.* negligible propagation time), the spatiotemporal contrast is given by (58), which we refer to as the pulse-limited case. For larger scattering volumes, this estimate is complemented by (66) and (68). Since all estimates are rigorous upper bounds, the smallest one takes preference. In particular, the contrast can only be pulse limited when all of 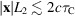, 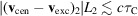 and 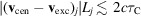 for 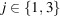 are satisfied. This is the case, irrespective of the scattering volume, when measuring at small scattering vectors 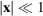 in the forward direction with respect to the excitation pulse, so that 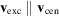. For all other cases, however, optimal (pulse-limited) contrast requires effectively that 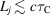 in all three dimensions. Importantly, we have that 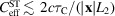 even in the best case.

Note that the scattering vector corresponds to a feature size *a* by 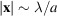. The constraint on 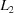 can thus be expressed as 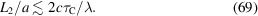
The right-hand side is on the order of 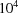 for inner-shell fluorescence.

### 4.3. Polarization effects   

In the preceding discussion we have assumed the emissions to be perfectly polarized such that they can be expressed as scalar functions 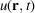. In an ensemble of emitters without a preferred spatial orientation however, the individual emitters produce (on average) *unpolarized* emissions. This further reduces the contrast.

Consider two fixed observation directions 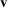 and 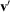. For any 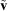 that lies in the plane spanned by 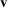 and 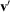 the emissions can be decomposed into two orthogonal polarization components, one parallel component 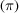 and one orthogonal component 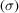. A detector that does not distinguish the polarization registers both components, 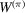 and 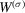, independently, such that the total deposited energy can be written as 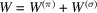. It follows that 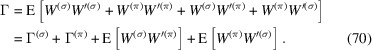
Assuming that the two polarization components are uncorrelated and equally intense, we obtain 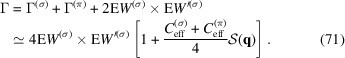
Comparison with (31) and using 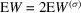 shows that effectively 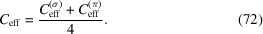
When both polarizations produce the same contrast, the contrast for unpolarized light is reduced by 2. On the other hand, when one component produces no contrast at all, the contrast is reduced by 4. We can thus incorporate unpolarized emissions simply by introducing 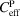, with 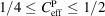, as an additional factor into (51).

## 5. Spatial sampling and finite detectors   

We have seen that the intensity correlation 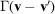 between two observation directions 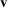 and 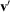 gives access to the structure factor 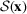 at the scattering vector 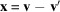. Next we shall discuss requirements on the sampling of observation directions in order to map the structure factor as a *function* of the scattering vector 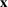. Note that we consider 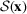 as the quantity of interest and ignore the topic of reconstructing the real-space emitter distribution. In particular, *resolution* exclusively refers to the resolution of 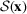. Moreover, we quantify and discuss how finite detectors influence the contrast.

### 5.1. Spatial spectrum of the correlation signal   

Set 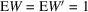 for this section. Consider 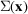 as a function of the unit-less scattering vector 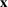. Its spatial spectrum is easily computed from (28) and can be expressed as 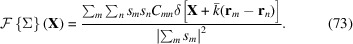
Note that the spectral amplitudes are real and positive since 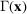 is real and symmetric by definition. The spectrum of the homogeneous background is a single δ function. The spectrum of Σ, on the other hand, is similar to the spatial self-correlation or generalized Patterson function (Cowley, 1995 ▸) of the emitter distribution, but attenuated by the 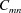 coefficients. The coefficients can be pulled out of the sums in the same way as in (31) by approximating them with 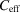, so that 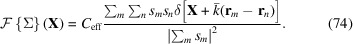
Integrating the spectrum over 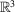 gives approximately 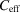.

Consider the previously introduced geometry, a homogeneous emitter distribution contained in a box with edge lengths 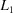, 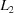 and 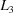 as shown in Fig. 3 ▸. Approximating the homogeneous emitter distribution by a continuous density, the spatial spectrum (73) can be expressed as 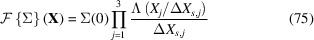
with 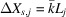, 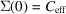 and the unit triangle function 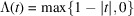.

More generally, *any* compact emitter distribution can be enclosed in such a box and its spatial frequencies are therefore limited by 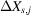 for all dimensions *j*. These frequencies only depend on the respective linear extent but not on the exact form of the scattering volume.

In other words, the structure factor and 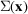 are *band limited* with bandwidth 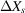. They can thus be sampled aliasing-free with the Nyquist rate 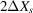, that is on a 3D grid with spacing less than or equal to 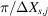.

### 5.2. Finite pixels   

The argument of 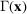 in (36) is determined by the difference of two unit vectors 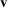 and 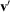, representing two observation directions. In a real system these observation directions are not points but finite solid angles, given by the pixel size and emitter-to-detector distance. We describe these solid angles as finite patches on the (normalized) Ewald sphere, *i.e.* 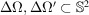. The measured correlation function can then be expressed as 
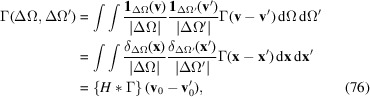
with 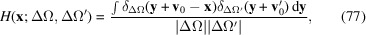
where 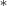 denotes a 3D convolution, 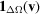 the indicator function of 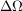 on the sphere 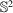 and 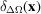 the Dirac surface delta function (Laplacian of the indicator) of 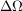 on 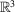. The vectors 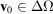 and 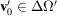 are the central directions of the two observation regions.

**Figure 4:**
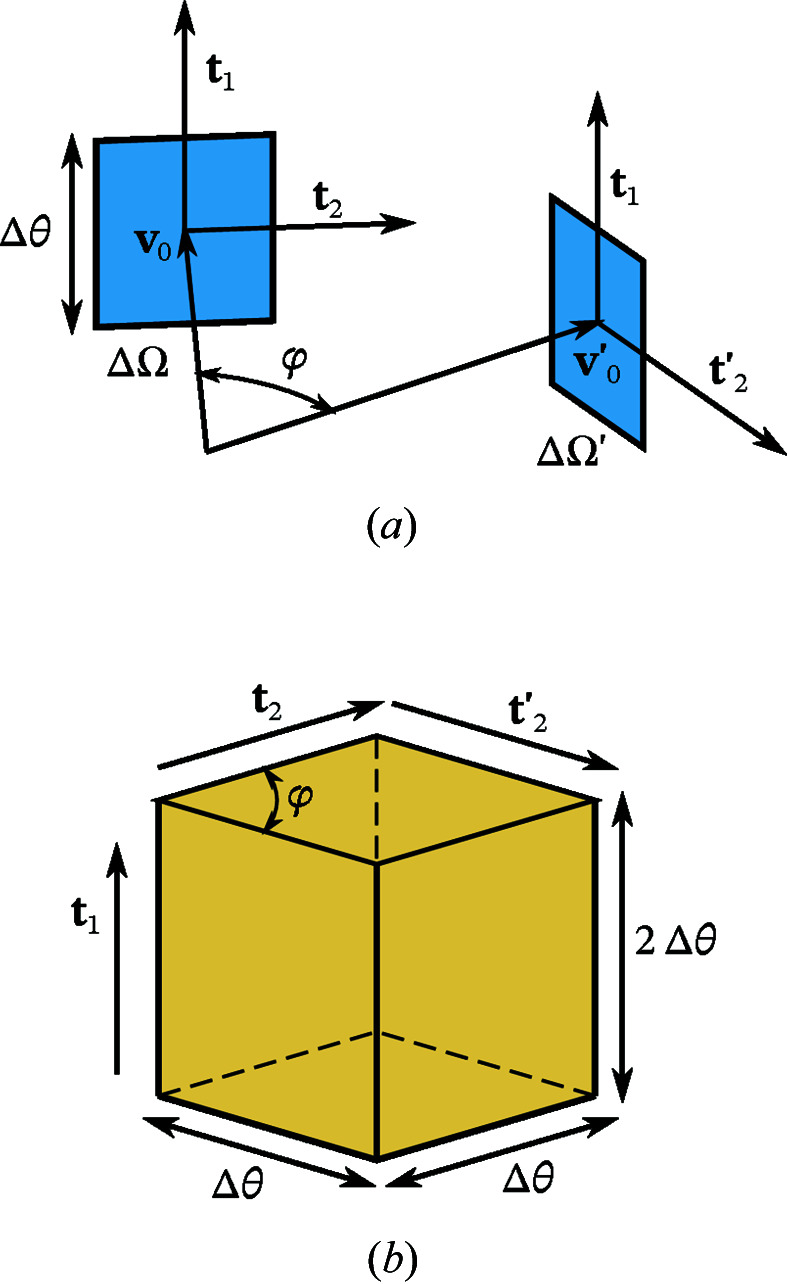
The effect of finite-sized detector pixels (*a*) on the correlations 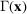 can be described by a 3D convolution 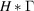. (*b*) Support of the convolution kernel *H*.

In practice, the two solid angles are small enough to neglect the curvature of the Ewald sphere. They can hence be approximated as planar patches with respective normals 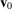 and 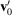 [see Fig. 4 ▸(*a*)]. We discuss the symmetric case of two partially aligned planar squares with relative angle φ and side lengths (in practice, the side lengths 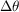 are given by the angular size of the detector pixels) 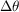 as sketched in Fig. 4 ▸(*a*). Here, the convolution kernel *H* can be computed analytically. However, the exact expression for *H* is less important than its support, which is sketched in Fig. 4 ▸(*b*). We approximate *H* by 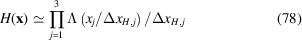
where 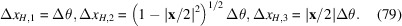
The Fourier transform of this approximate kernel reads 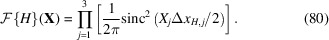
As expected, the finite pixels cause a low-pass filtering of 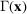 with angular frequency scale 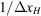 and thereby limit the resolution with which 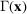 can be measured, irrespective of the sampling rate. The more general case of unaligned detectors corresponds to a less regular convolution kernel but qualitatively has the same effect.

### 5.3. Contrast   

Finite pixels limit the resolution with which the structure factor 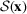 can be measured since higher spatial frequencies are attenuated. However, due to the presence of the intrinsic background, they also limit the achievable intrinsic contrast. We will illustrate this with a simple example that can be computed analytically: the homogeneous emitter distribution in a box as shown in Fig. 3 ▸.

For that purpose we decompose 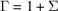 into a sum of constant unit background and signal 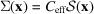. The measured correlation becomes 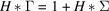, using that the background is unaffected by the convolution. We may factorize the approximate convolution kernel 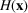 = 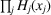 as well as the signal 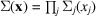 into 1D functions, such that 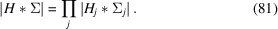
Each factor satisfies the inequality 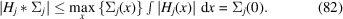
Exploiting the Fourier convolution theorem we find a second triple of inequalities,^2^

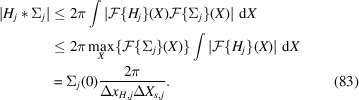
Combining the two inequalities yields 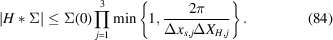
Substituting 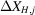 and 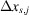 shows 
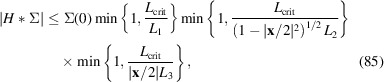
with 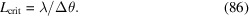
A generalization of this result, not relying on specific assumptions on the shape of Σ and *H*, is presented in Appendix *A* [App appa]. Equation (85) implies that the contrast falls off with 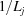 for each linear extent 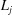 of the scattering volume that exceeds a critical length in the order of 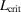. Note that (85) is based on a not necessarily sharp upper bound on the signal strength, so that the contrast may suffer even for smaller 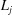.

To put this into perspective, consider *K*α radiation of iron (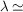 0.19 nm) and a typical pixel detector with 50 µm pixel size. Placing the detector 10 cm, 1 m or 10 m from the emitters corresponds to 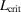 of 0.38 µm, 3.8 µm or 38 µm, respectively.

## 6. Photon count estimates   

We have seen in (48) that the SNR is bounded by an expression proportional to 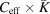. Here, we derive universal upper bounds on this product, based on the fact that the emitter density 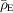 is finite and that the contrast decreases with increasing extent of the scattering volume.

The mean number of photons emitted into 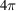 from a cuboid scattering volume with edge lengths 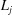 (see Fig. 3 ▸) is 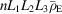, where *n* is the emission efficiency in the considered energy band. A detector pixel of angular size 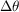 and quantum efficiency η registers on average 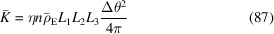
counts if we neglect self-absorption within the scattering volume. We have derived two independent constraints between scattering volume and contrast. First, (66) implies, in particular, that the contrast falls off with 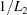 for 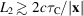 (coherence time constraint). We set 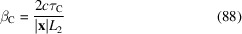
to quantify the margin by which the coherence time constraint is satisfied. Second, (85) implies that the contrast falls off with 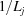 for each dimension *j* in which the linear extent of the scattering volume 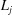 is larger than a certain critical size (sampling constraint). We analogously set 
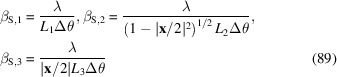
to quantify the margin by which the sampling constraints are satisfied. Inserting (88) and (89) into (87) yields 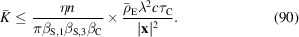
The unit-less scattering vector corresponds to a feature size *a* via 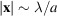, so that (90) can be expressed as 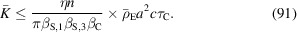
Having expressed 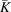 in terms of the parameters β, we can optimize the product 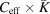. Equations (66) and (85) imply that 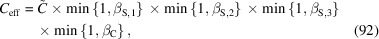
where 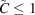. Since (92) becomes proportional to every β that is less than one, it follows that 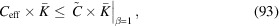
where 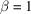 refers to all the coefficients. In particular, increasing the mean photon count 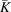 beyond its value for 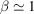 does not increase the SNR, because the contrast is reduced just as much. In other words, there is an optimal size of the scattering volume, beyond which the SNR does not increase any further.

Although some β-dependent factors are included in 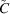 and are not explicitly written in (92), they only reduce the contrast further. Because of these factors and since the individual inequalities are not sharp (there can be significant margins between the left- and right-hand side), the maximum SNR may actually be attained with some 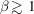 but, nevertheless, will not exceed the given bound.

For 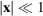, the sampling constraint on 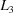 becomes arbitrarily large and thereby exceeds the self-absorption length (the 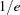 length of the emitted light in the material), so that (90) strongly overestimates the achievable 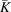. We can use 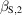 instead of 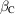 to quantify 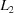 to obtain 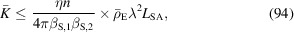
where 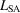 is the self-absorption length. This estimate is independent of the coherence time.

To put (91) into perspective, consider a dense mono-elemental iron crystal with 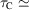 2.6 fs, *a* ≃ 0.29 nm and 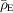 ≃ 85 nm^−3^. For these values, (69) yields 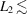 2.3 µm. Optimistically assuming 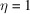, 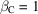 and 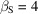, (91) shows that 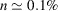 of all atoms are required to emit a photon for an average photon count of 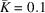 photons per detector pixel.

The right-hand side of (91) is proportional to the squared feature size 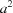 and therefore grows quadratically with increasing *a*. However, when measuring atomic distances, it effectively scales with 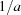, because the mean emitter density 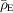 scales with 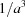. Consider a crystal structure with lattice constant *a*, such that 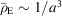. The number 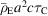, which bounds the mean photon count 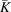 in (91), is then essentially governed by 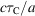. In general, increasing the feature size *a* increases the SNR only as long as the mean emitter density decays slower than 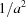.

As an example consider a crystal with ten times the lattice constant of iron, 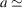 2.9 nm, and one iron atom per unit cell. The emission efficiency needs to be ten times as high for the same photon yield as for the pure iron crystal, so that, based on our previous estimate, 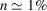 for 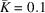.

In summary, we have shown that an optimal size of the scattering volume exists. Larger volumes do not result in higher SNR, even though the photon count is increased. The mean photon count corresponding to optimal SNR can be estimated by (90), (91) or (94), with all coefficients 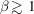.

## 7. Discussion   

Next, we use our findings to discuss the feasibility of different experiments and to highlight the challenges. We chose two particular experiments to illustrate the fundamentally different experimental regimes.

### 7.1. Determination of illumination spot size   

First, consider an experiment to determine the illumination spot size, following Inoue *et al.* (2019 ▸). A spot size of 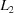 = 0.5 µm with copper *K*α radiation (λ = 0.154 nm) corresponds to a scattering vector 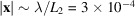. Correspondingly, the width of the zeroth-order correlation peak has to be resolved, similar to the primary beam profile in classical small-angle X-ray scattering geometry. To this end, the detector is placed in the forward direction a few metres downstream of the sample. The sample can be a foil so that the focal spot size of the excitation pulse determines the transverse extent of the scattering volume. Here, since the scattering vectors are small, the sample thickness 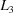 can be chosen largely independently of the detection geometry, in practice up to the self-absorption depth. In particular, the contrast is pulse limited, because 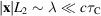 and 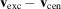 vanishes.

As a rough estimate, consider a 10 fs excitation pulse, 2 fs coherence time (corresponding to copper *K*α), 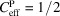 and 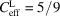 (the two *K*α lines) so that 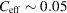. The self-absorption length depends on the material and the emission line. For the *K*α lines of copper with 8.96 g cm^−3^ mass density the 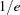 length is 22 µm. Moreover, by using a highly focused XFEL beam it should be possible to ionize a large fraction of the sample atoms, so that a mean photon number of 0.1 per pixel can be easily achieved according to (94). Using a pixel detector with 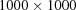 pixels provides 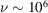 parallel realizations. Inserting these numbers into (49) estimates that at least 14 pulses would be required to sample the half maximum 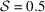 with a signal-to-noise level of 10. The experiment is relatively robust, because translations and rotations of the scattering volume do not affect the signal in first order. Inoue *et al.* (2019 ▸) used such a measurement to determine the pulse duration and the focal size at SACLA. In contrast, if one wanted to perform an analogous experiment at a laser-driven plasma source (Schoenlein *et al.*, 2019 ▸), where on the order of 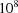 copper *K*α emissions occur per excitation pulse of about 100 fs, the emission efficiency can be estimated to 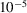 based on the atom density of copper, a spot size of about 2 µm and a target thickness of 10 µm. The low emission efficiency results in at most 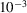 photons per pixel according to (94). The squared dependence on the photon count and contrast in (49) then would require more than 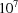 pulses. Ironically, initial attempts of such an endeavor had motivated the present work.

### 7.2. Atomic resolution from Bragg scattering   

Second, consider an experiment to resolve crystal planes. As an example, consider the 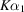 radiation from iron with 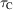 = 2.6 fs, λ = 0.19 nm and a total *K*-shell fluorescence yield of 35% (Schoonjans *et al.*, 2011 ▸). Pure iron at room temperature has a b.c.c. (body-centered cubic) lattice with lattice constant 0.29 nm and number density 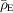 = 85 nm^−3^. Since the wavelength is fixed by the emission line, the lowest (110) reflections have a (unit-less) scattering vector of 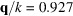. To reach this peak, the pixel array detector needs a field of view of about 55° in one dimension. The accessible scattering vectors are bounded by 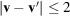, so that the highest accessible reflection is the (220). As previously discussed, the coherence time constrains the sample size to about ≃2 µm through (69).

Consider a cubic perfect crystal with diameter 0.5 µm (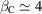) so that the contrast is excitation-pulse limited. The sampling constraint with 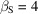 requires an angular pixel size of 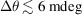, so that a detector covering 60° requires 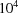 pixel-columns in that direction. This could be realized by arranging ten 1M detectors in an arc. Fewer detector modules with larger gaps could also be used in principle, at the cost of covering only specific Bragg peaks.

A very optimistic estimate on the contrast for a 10 fs-long XFEL pulse gives 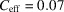, for perfect conditions. This estimate assumes a perfect single crystal and that the system is perfectly stationary and stable. In particular, the orientation of the sample has to be stable on the order of magnitude of 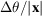. An uncertainty σ in the sample orientation that exceeds this limit smears out the Bragg peaks over several resolution elements and therefore decreases the contrast by 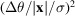. An uncertainty of 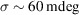, for example, would decrease the contrast by 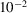 down to 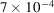. Whereas a stable sample orientation of 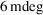 is easily obtained for static and extended samples, it is non-trivial to reach this accuracy in single-particle experiments with random orientations. The coherent diffraction signal of the particles could be used to calculate the particle orientation in each pulse. However, solely from a sampling perspective, detectors with a huge pixel number would be necessary to reach the desired angular accuracy σ, neglecting further experimental inaccuracies of 3D orientation determination. In this specific example, the coherent diffraction signal needs to be sampled on a detector with a minimum of about 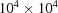 pixels to obtain 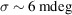. Note that additional factors such as lattice vibrations (Debye–Waller factor), lattice strain and defects have not been considered.

Next, we discuss the multiplicity of correlation measurements from a single pulse (detector frame). The scattering vectors given by the sets of all pixel pairs are distributed in a volume (Classen *et al.*, 2017 ▸). Most of the realizations correspond to small scattering vectors, while the larger scattering vectors of the Bragg peaks have a strongly reduced multiplicity ν. Assuming 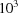 pixel rows gives a conservative estimate of 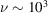 parallel realizations for a Bragg peak signal.

Assuming a mean photon count of 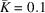 and using (49) we see that on the order of 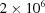 realizations are required for an SNR of 10, which could be acquired within 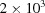 pulses, assuming optimal conditions. Since the number of pulses depends quadratically on the contrast, an uncertainty in the sample orientation of 0.06° would increase the required number of pulses to 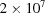, which is a sizeable number for a dense mono-elemental iron sample.

It will be challenging to realize a mean photon count of even 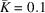 for dilute samples. Using (91) with 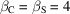 and assuming 100% detection efficiency shows that about 0.4% of the atoms in the pure iron sample need to emit a *K*α photon for a per-pixel photon yield of 0.1. Correspondingly, to achieve the same resolution in a sample with 1% iron content, 40% of the iron atoms would have to emit a *K*α photon, which is already above the *K*-shell fluorescence yield. Therefore, such dilute samples can only produce a photon count of less than 0.1 photons per pixel, even when fully ionized. Optimizing the geometry for smaller scattering vectors, *i.e.* coarser resolution, enables one to use larger scattering volumes and can improve the photon yield to some degree. However, the given estimate already corresponds to a sample with diameter 0.5 µm, which does not leave much room for increase in the case of nanocrystallography or single-molecule diffraction.

## 8. Summary and conclusions   

We have derived comprehensive equations relating the two-point intensity correlations to the structure factor 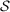 of the emitter configuration. We have reproduced the expression given by Classen *et al.* (2017 ▸) up to an additive term of order 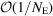, which is hence irrelevant for a large number of atomic emitters. This agreement with the results of Classen *et al.* (2017 ▸) and the classical description presented by Trost *et al.* (2020 ▸) underlines that IDI does neither rely on any non-classical states of light nor beat any classical limits.

By including time dependence, we have obtained an explicit expression for the contrast between the structural signal and the inherent background in the correlation functions. Equation (28) shows that the total signal can be decomposed into a sum of terms from individual pairs of emitters and that each term is attenuated by a coupling constant 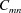 that takes values from 0 to 1. Averaging the coupling constant over all pairs of emitters gives an effective contrast 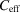 of less than one.

We have given an estimate for the SNR, equation (48). In the low-photon regime, it scales linearly with the mean photon count 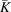 and the signal strength 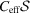. Importantly, we have obtained a rigorous upper bound on the SNR for two statistically *dependent* coincident measurements.

Based on our model, we have identified several factors influencing the contrast and have quantified them in terms of experimentally accessible parameters. First, the fact that multiple emission lines contribute to the total signal decreases the contrast by a factor 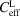 that is inversely proportional to the number of emission lines and depends on their relative strengths. This emphasizes the need for an effective energy discrimination of the emitted radiation. Second, the lack of polarization of the emitted radiation decreases the contrast by a factor of 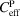, where 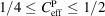 depending on the angular separation of the two observation directions. Third, the finite coherence time 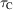, the finite propagation time through the sample, and the finite duration of the excitation pulse 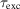 strongly affect the contrast by a spatiotemporal factor 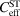. The scaling of the contrast depends on the relative magnitude of the three involved time scales. In particular, the pulse-limited scaling, (58), does only apply for small scattering volumes, or when both observation directions are in a small cone around the propagation direction of the excitation pulse 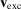. In general, the contrast is additionally affected by the extent of the scattering volume, as detailed in (66). It implies that 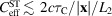 which effectively restricts the linear extent 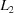 of the scattering volume for larger scattering vectors 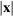. Although for two fixed directions (or pixel coordinates), the size restriction applies to only one dimension, for effective use of a 2D pixel array, involving simultaneous measurements at many different scattering vectors 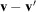, it effectively applies to all dimensions.

The contrast is also affected by the integration associated with finite detector pixels. If the detector pixels are too large to properly resolve the speckle patterns, the signal is decreased while the inherent background remains unaffected, resulting in a loss of contrast. The scaling of the contrast can be expressed in terms of a critical length 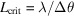 depending on the wavelength λ and the angular pixel size 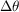. According to (85), the contrast is decreased proportional to 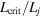 for each dimension *j* in which the diameter of the scattering volume exceeds a critical length. For small scattering vectors, only the transverse dimensions 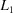 and 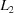 are relevant while the thickness 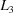 is effectively unconstrained, whereas for large scattering vectors, all dimensions contribute approximately equally.

Importantly, the constraints of the critical linear extent of the scattering volume, as discussed above, also directly limit the total photon yield per pixel for SPE due to their finite number density 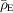. We have shown in particular that the SNR cannot be improved beyond an optimal value, which is significantly lower than anticipated. Increasing the photon count beyond its corresponding optimum decreases the contrast and does not improve the SNR. The optimal photon count and SNR depend on the magnitude of the scattering vectors 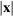 and thus on the probed length scales. For small scattering vectors, the sample thickness can be increased independently of the transverse size to optimize the photon yield, so that the photon yield is bounded by (94). In contrast, for larger scattering vectors, all dimensions enter with the same scaling and the photon yield is bounded by (91). Although both constraints had been mentioned by Classen *et al.* (2017 ▸) and Trost *et al.* (2020 ▸), their implications for the photon yield had not yet been quantified or discussed.

Based on these bounds, we have discussed examples of two fundamentally different experimental regimes. First, an experiment aiming to resolve the geometry of the scattering volume as demonstrated by Inoue *et al.* (2019 ▸). Second, an experiment to resolve the crystal structure within the scattering volume as was proposed by Classen *et al.* (2017 ▸). For the latter case, we have shown that the best possible SNR is inversely proportional to the lattice constant when aiming for atomic resolution. We have also given estimates for the best possible photon count and the corresponding fractions of ionized atoms. Moreover, we have discussed how the pulse-to-pulse orientational stability influences the SNR.

We would like to stress that the simulation presented by Classen *et al.* (2017 ▸) strongly overestimates the achievable photon count and SNR for the discussed geometry. More than 5 photons per pixel are only possible with a large and dense sample with optimal geometry, which is inconsistent with the stated assumptions. Similarly, Trost *et al.* (2020 ▸) used mean photon counts in the range from 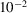 to 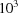 in their simulations. We have shown that for atomic resolution IDI even 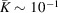 can be achieved without sacrificing the SNR only for samples with a high density of emitters, whereas for dilute samples with a lower emitter density, such as macromolecules, 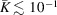 is more realistic.

In light of the low SNR, even under idealized conditions, and the required pulse-to-pulse stability, we come to the conclusion that utilizing IDI for serial crystallography will be extremely challenging in general – even more so for diffractive imaging of single molecules, as was envisioned by Classen *et al.* (2017 ▸) and deemed achievable by Trost *et al.* (2020 ▸).

We hope that our quantitative estimates may serve as a solid basis for discussing the use of structure determination based on incoherent emissions. In particular, we hope that our results may be useful to assess the limit of length scales that can be reasonably probed. Finally, we would like to mention that the derived limits, which are quite fundamental, scale very favorably with the coherence time of the emissions. It should therefore not escape our attention that emissions with longer coherence time such as visible light fluorescence could result in quite realistic IDI experiments on larger length scales.

## Footnotes

1 Here we leave aside effects due to secondary scattering of the emitted photons such as for Kossel lines (Kossel *et al.*, 1935 ▸; Laue, 1935 ▸) or fluorescence holography (Gog *et al.*, 1996 ▸), which do carry some information on the structure hosting the fluorescent emitters.

2 Here we implicitly approximate the discrete emitter distribution by a smooth density, to keep the derivation short. A more general derivation is discussed in Appendix *A* [App appa].

## Appendix A Contrast for arbitrary emitter distributions

We utilized concrete assumptions on the shape of the functions Σ and *H* in the derivation of (85). In particular we assumed the multiplicative separability of the two functions to estimate each dimension separately. Clearly, this strategy fails for more general classes of Σ. Here we outline a similar derivation without the need the factorize Σ and *H*.

Following the same approach with the full 3D functions shows that  and

Unfortunately the next inequality in (83) cannot be applied here, because  is a distribution. Nevertheless,  is smooth and slowly oscillating because *H* is compactly supported. We may therefore approximate the emitter distribution  by a continuous emitter density  such that

We can thus express the Fourier transform of Σ by the normalized cross-correlation of ,

Substituting  in (95) gives

We obtain

where  denotes the volume of the emitter distribution [in units of ] and

Our approximate expression (78) yields  = , roughly corresponding to the volume of the support of *H*. Equation (99) is a generalization of (83) for arbitrary detector shapes and scattering volumes.

Approximating  by a continuous emitter density requires  to be approximately constant on the (dimension-less) length scale of the emitter distance. On the other hand, in the limit of very wide *H* and thus very sharply peaked  the individual δ-distributions are isolated in (95). We then obtain that

independent of the scattering volume and detector size.

## Appendix B Variance of dependent random variables

Let  be a pair of possibly dependent random variables on . Define  conditionally via  as a tensor product of Poisson distributions. We seek an expression for  in terms of *X* and .

We have  and , as well as

The second moment can be written as

where the last step can be shown as follows. Let  be two non-negative numbers and define  . Then

The second *central* moment then becomes

which can be expressed as

## Appendix C List of the main symbols used in the paper

, number of emitters.

, position of emitter *m*.

, , random variable and probability that emitter *m* emits a photon.

, photon energy of the emission from emitter *m*.

, time when emitter *m* emits a photon.

, scalar field amplitude of the emission from emitter *m*.

, energy flow of  into the solid angle .

, total scalar field amplitude from all emissions.

, total energy flow into the solid angle .

Γ, two-point correlation of energy flows (or photon counts).

Σ, two-point covariance of energy flows (or photon counts).

, complex degree of coherence (CDC).

, coherence time.

, coupling coefficients of the two-photon contributions.

, average coupling coefficient/contrast.

, , observation directions.

, unit-less scattering vector.

, structure factor.

*K*, photon count (random variable).

, average photon count (number).

*R*, number of independent realizations.

, multiplicity/number of realizations for scattering vector .

, distance from emitter *m* to *n*.

, difference of emission times.

, probability density of .

, cycle-averaged intensity of the excitation pulse.

, temporal autocorrelation of the excitation pulse .

, propagation direction of the excitation pulse.

, duration of the excitation pulse.

, arithmetic mean of observation directions.

, , side lengths of scattering volume.

*a*, feature size or lattice constant.

, angular pixel size.

*c*, speed of light.

, mean emitter density in the scattering volume.

λ, (mean) wavelength of the emitted light.

η, detector quantum efficiency.

*n*, effective emission efficiency.

, number that quantifies the coherence time constraint.

, numbers that quantify the sampling constraints.
